# National Trends in Cycling in Light of the Norwegian Bike Traffic Index

**DOI:** 10.3390/ijerph18126198

**Published:** 2021-06-08

**Authors:** Solveig Nordengen, Lars Bo Andersen, Amund Riiser, Ane K. Solbraa

**Affiliations:** 1 Department of Sport, Food and Natural Sciences, Western Norway University of Applied Sciences, 6851 Sogndal, Norway; lars.bo.andersen@hvl.no (L.B.A.); amund.riiser@hvl.no (A.R.); ane.solbraa@hvl.no (A.K.S.); 2 Department of Sports Medicine, Norwegian School of Sports Science, 0806 Oslo, Norway

**Keywords:** bicycle transport, employee commuting, monitoring bicycle employee ride, the Norwegian bike traffic index, active travel

## Abstract

National and international strategies and recommendations are intended to increase physical activity in the general population. Active transportation is included in interdisciplinary strategies to meet these recommendations. Cycling seems to be more health enhancing than walking for transportation since cycling seems to reduce the risk of cardiovascular disease and associated risk factors. Furthermore, the health benefits of cycling are proven to outrun the risk of injuries and mortality. Politicians seem to approve costly infrastructure strategies to increase the amount of cycling in the population to improve public health and shift to more sustainable travel habits. A linear relationship between cycle-friendly infrastructure and the amount of commuter cycling has been demonstrated. However, in Norway and on a global level, there is a lack of robust evaluations of actions and sensitive monitoring systems to observe possible change. Therefore, we aimed to develop the Norwegian bike traffic index and describe the national, regional, and local trends in counted cycle trips. We used a transparent methodology so that the index can be used, developed, and adapted in other countries. We included 89 stationary counters from the whole country. Counters monitored cycling from 2018 onward. The index is organized at local, regional, and national levels. Furthermore, the index is adjusted for population density at the counter level and presented as ratio of counted cycle trips, comparing 2018 to subsequent years. The index is presented as a percentage change with 95% confidence intervals. In Norway, counted cycle trips increased by 11% from 2018 (100, 100–100) to 2020 (111.0, 106.2–115.1), with large geographical differences. In Southern Norway, there was a significant increase of 23%, and in Northern Norway, there was a nonsignificant decrease by 8% from 2018 to 2020. The indices may indicate possible related effects of local to national cycling strategies and how the COVID-19 pandemic has affected Norwegian travel habits in urban areas.

## 1. Introduction

Official Norwegian strategies and recommendations are intended to increase physical activity in the population [1,2] and highlight the necessity for interdisciplinary strategies that include active transportation (e.g., cycling). Cycling is associated with reduced risk of type 2 diabetes [3], cancer [4,5], and all-cause mortality [4,5]. Cycling further mitigates the risk factor profile for cardiovascular disease (CVD) [6] and lowers the risk for CVD incidence and CVD mortality in both men and women [7]. A dose–response relationship between cycling and all-cause mortality has been observed [8], and any cycling is recommended. The health benefits of cycling have been observed to be 21 times higher than the risk of injuries and 238 times higher than the risk of mortality alone [9], and the economic benefit is five times larger than the cost of building new cycle infrastructure [10,11].

The Norwegian Public Road Administration (NPRA) launched their national strategy for cycling in 2012 [10]. This strategy acts as a base document for the National Transport Plan 2014–2023, highlights the need for increased use of cycling as a mode of transportation, and is continued in the latest national transport strategy [12]. The primary objective of the national strategy is to increase the number of trips by bicycle to 8% at a national level by 2023. In addition, the strategy aims to reach 80% commuter cycling for children traveling to school, promote cycling as a transportation mode choice, double the usage of bicycles in high-density cities and municipalities, and increase safety and bikeability [13]. However, since the 1990s the total number of cycling trips has decreased from 7 to 4% in Norway as reported by the national travel survey (RVU) [12]. The number of total trips is low taking into consideration that 80% of the population has access to a bicycle [12].

The national strategies for active transportation and the increased interest in and attention paid to cyclists have resulted in projects such as the Førde Package [14]. In 2012, Førde Municipality signed an agreement with the NPRA and Sogn og Fjordane County Authority to become a ‘cycle city’. The aim of this agreement is to ‘increase bicycle use, among other things by transferring transportation from private cars to cycling’. To increase sustainable commuting, the road network in Førde Municipality will be upgraded for EUR 154 million through the Førde Package. This package includes constructing new infrastructure for cycling and walking during a period of 8 years that began in October 2016. The Førde Package’s master plan is comprised of 20 interventions, including separate bike lanes, shared lanes with walkers, shared lanes with drivers, and cycle roads.

The approval of the Førde Package underpins the fact that policymakers seem particularly keen to increase the number of cyclists since cycling allows for fast and efficient urban travel, requires minimal space for tracks and parking, and causes no air or noise pollution [15,16]. Infrastructure interventions have shown promising effects on the number of cyclists [17,18,19], and cycle-friendly infrastructure has a strong association with the number of cyclists with a coefficient of determination (r^2^) from 0.3 to 0.8 [11,16,19]. The relationship seems to be stronger in larger cities than in smaller ones [11]. In Europe, a linear relationship between metres of cycle-friendly infrastructure per citizen and cycling has been reported [11]. Although cycle-friendly infrastructure is important when attempting to increase the number of cyclists, infrastructure alone is rarely sufficient [20]. There is a need for robust scientific evaluation of infrastructure interventions and how interventions in the built environment influence cycling habits within population groups [21,22]. A bike traffic index organized at different levels (i.e., the regional and national level), such as the Danish bike traffic index [23], may provide a reference point and be helpful for municipalities wanting to evaluate cycling-specific public health goals [24]. A bike traffic index based on bicycle counters may be more valid than surveys since it reflects the actual number of counted cycle trips independent of residence, age, or recall bias [23]. Furthermore, when the index is based on continuous counting results, the model is sensitive to actual changes [23].

Therefore, we aimed to develop the Norwegian bike traffic index and describe the national, regional, and local trends in counted cycle trips. The bike traffic index will be of local, regional, national, and even global interest since it describes the baseline number of counted cycle trips in Norway and provides a transparent method and adaptable index which monitors trends and possible related effects of local to national cycling strategies.

## 2. Methods and Accuracy

### 2.1. Bike Traffic Data

Coordinates, number of passing cycle trips, coverage (percentage of valid days for a bicycle counter), and first operative day of the bicycle counters were derived from www.trafikkdata.no; accessed on 1 August 2020, which contains data under the Norwegian license for open government data distributed by the NPRA. In addition, the indices were based on data distributed by Statistics Norway. The daily traffic is the sum of valid counted cycle trips. The daily traffic value has consecutive coverage. Coverage is a measure of the amount of data with sufficient quality (operative more than 95% of the time), where low coverage indicates low representativeness while high coverage indicates high representativeness.

### 2.2. Population Density

Population density was investigated in a geographical information system (QGIS version 3.10.3–A coruña, Free Software Foundation, Inc., Boston, MA, USA), which we used to investigate the number of individuals living within a 5-km grid where a counter was located. The static grid was a network of evenly spaced horizontal and vertical lines covering the whole country. The present layer was a horizontal and vertical grid network of 5 × 5 km where counters were placed at any point within the grid. Thus, we reported the total number of people living within a grid where a counter occurred. To locate the counters, we firstly recoded the coordinates of the counters as X and Y values for longitude and latitude, respectively. Second, we imported information about the population density by using Statistics Norway’s 2019 defined raster file with a 5-km grid size downloaded from www.geonorge.no (accessed on 1 August 2020). To calculate the population, we summarized the number of people living in a grid with an included counter. Furthermore, we divided the proportion of individuals living in a grid and the number of individuals within a counter’s grid by the total number of individuals living within a grid with a counter.

### 2.3. Included Counters

**Figure 1:**
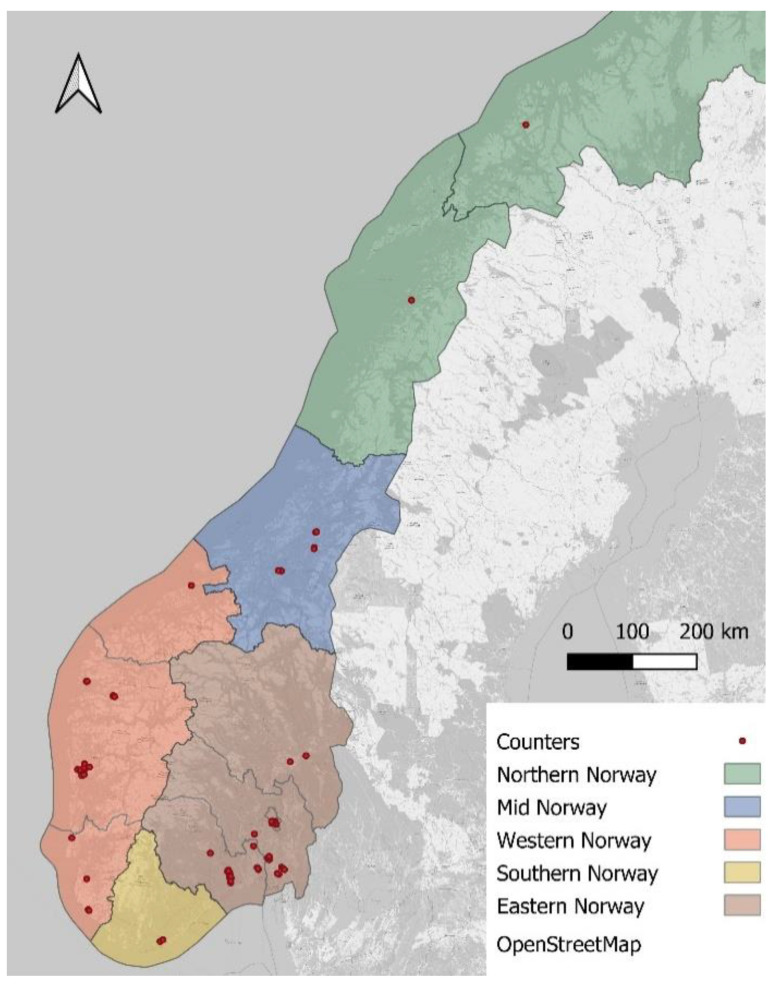
Location of included counters and regional areas.

In total, we included 89 stationary counters in the bike traffic index (Figure 1). All included counters have been operative since 1 January 2018. We identified 25 local areas with a minimum of one operative counter. Each local area is presented as local indices. The number of counters included in the local indices ranged from 1 to 14 with a median of 2. The mean population density within the local indices ranged from 840 to 93,176 individuals (see Table 1 for the number of counters and mean population density within the local index). The local indices were further located in an appropriate region, which was either Northern, Mid, Western, Southern, or Eastern Norway. The mean population density within the regions ranged from 15,148 individuals to 29,670 individuals, and the number of counters ranged from 3 to 48 (see Table 1 for details). Eastern Norway contained 54% of the included counters and included the local index with the highest mean population density.

### 2.4. The Counters

The included counters were either inductive loop monitors (83%) or piezoelectric counters (17%) and classified vehicles passing. An inductive loop is a detection system that senses metal objects that pass over the in-ground ‘loop’ [25], and piezoelectric counters generate a count when the material is physically deformed [26]. The monitors provided a timestamp, direction, and speed for the object passing. When automatic and manual observations are compared, inductive loop and piezoelectric monitors have previously demonstrated high accuracy and correlation with Pearson’s r = 0.99 and 1.00, respectively [26]. A 1.7 to 2.7% underestimation of counted trips for inductive loop monitors and piezoelectric counters has previously been detected [26]. When tested, the monitor has managed on average 128 to 129 (283 to 355 maximum) counted trips per hour [26].

### 2.5. Missing Data

When daily traffic had coverage of less than 95%, the data was set to missing (user-missing). Throughout the years 2018, 2019, and 2020, there was a total of 6% missing data days. System- or user-missing data were replaced by linear interpolation as missing data were replaced by the mean of the last value before the missing value and the first valid value after the missing value. There were both single days and longer periods (weeks) of missing data. Reasons for system-missing data may be error on the counter, construction on site, ice on the ground, or weather. When missing data occurred in 2020 with no valid value after the period of missing values, the data were registered as missing. Following the procedure by NPRA [27], successive data were deleted in the comparable month (i.e., if there were no valid data for December 2020, data for December 2018 were deleted).

### 2.6. Traffic Pattern

Bike traffic may be categorized as commuter cycling or recreational cycling [24]. Commuter cycling is mainly cycling done as a mean of transportation [24]. Recreational cycling is cycling done for leisure, social, or fitness activities [24]. Miranda-Moreno et al. [24] argue that this may be oversimplified because the characteristics differ between weekdays and weekends and because the traffic volume depends on location rather than facility types. Following Minge et al.’s adapted methods [25], we calculated two indices for a random week for each counter. The first index is a relative index of weekend versus weekday traffic (*WWI*; 1). The second index is a relative index of morning (7:00–9:00 a.m.) to midday (11:00 a.m.–1:00 p.m.) traffic (*AMI*; 2).

(1) $$WWI=\frac{V_{we}}{V_{wd}}$$

where *WWI* = weekend/weekday index, *V_we_* = average weekend daily traffic, and *V_wd_* = average weekday daily traffic.

(2) $$AMI=\frac{\sum_{7}^{8}\;V_{h}}{\sum_{11\;}^{12}V_{h}}$$

where *AMI* = average morning/midday index, *V_h_* = average weekday hourly count for hour (h), and hours are given as the starting time of the hour.

The traffic pattern is classified as commuter cycling when weekday traffic is higher than weekend traffic (*WWI* > 1) and the weekday hourly pattern is commuter-like with more traffic in the morning than at midday. The traffic pattern is multipurpose when weekend traffic is higher and weekday hourly patterns are not commute-like. Commute-mixed is when weekday traffic is higher than weekend traffic but weekday hourly patterns do not indicate typical commuting. Finally, a multipurpose-mixed traffic pattern is when weekend traffic is higher although weekday hourly patterns are indicative of commuting (*AMI* > 1). Among the 89 included counters, 75 (85%) were defined as ‘commute’, 11 (12%) as ‘commute-mixed’, and 3 (3%) as ‘multipurpose-mixed’.

### 2.7. Principle of the Index

The inspiration for the Norwegian bike traffic index came from the Danish bike traffic index [23]. Simply put, the Norwegian index is a ratio of counted cycle trips between two successive years:

(3) $$R=\left(\frac{Y}{X}\right)100\;\%$$

where *R* is the ratio of *Y*—the year compared to the baseline year, *X*—multiplied by 100%. The baseline year is thus set to 100%, and we can follow a percentage change between years *X* and *Y*.

The index is organized at three different levels: local, regional, and national. The local index is adjusted for population density at the counter level. The local index is a sum of annual counted trips from each counter. By this method, the changes in the model mainly affect the local index. Separately, the local index is an uncertain measure with a large confidence interval due to the low number of counters [23] and therefore must be interpreted with caution. Furthermore, the regional indices and the national index are the weighted sum (counted trips multiplied by the proportion of residents at the counter level) of all trips in the region or country.

The indices are both presented as index based on annual counts and as monthly average daily traffic. For annual counts, 2018 is set as the baseline year, and successive years are thus compared with the baseline year.

### 2.8. Calculation of Confidence Intervals for Traffic Indices

We calculated confidence intervals for the traffic indices according to the directions of the NPRA [27]. This approach is based on paired sets of valid data for the period in question and for the reference year, respectively, at each site of interest and for each period (e.g., hour, day, month). We calculated a variance for all valid pairs of data. More specifically, for each site, we calculated and squared the difference between the index of the site and the average for the whole country (or region or local area). We weighted the squared difference in proportion to the traffic volume and calculated a correction to account for using estimated parameters rather than the true (but unknown) value. This last correction corresponds to dividing by (*n* – 1) rather than by *n* when calculating the common variance from *n* different independent values with equal weight, producing an unbiased estimate of the true but unknown variance. The standard deviation is taken as the square root of the calculated variance.

(4) $$s_{a,p,y}=\sqrt{\sum_{i=1}^{n}\;\left[\frac{N_{i,p,y_{0}}}{N_{a,p,y_{0}}}{\left(Q_{i,p,y}-Q_{a,p,y}\right)}^{2}\right]\cdot \;{\left[1-\sum_{i=1}^{n}{\left(\frac{N_{i,p,y_{0}}}{N_{a,p,y_{0}}}\right)}^{2}\right]}^{-1}}$$

Here, *n* denotes the total number of counted cycle trips, and *i* is a running variable for sites 1, 2, …, *n* within area a (the whole country, region, or local area). *p* is the period in question (hour, day, month, year), which for the present case is a full year. *y* is the year in question (2019 or 2020 for the present case), and *y*_0_ is the reference year (2018 for the present case). *Q* denotes an index, meaning the ratio of the recorded traffic for two different years. Thus, *Q_i_*_,*p*,*y*_ denotes the ratio between the counted cycle trips at site *i* during period *p* at year y and the corresponding counted trips at the same site and period in the reference year *y*_0_. Referring to the squared term in the numerator, if the indices for all sites within an area are equal (and equal to that of the average of the whole area), the standard deviation is zero. If the indices differ much between sites, and there are thus many large deviations from the area mean index, the standard deviation will increase correspondingly.

This is the standard deviation of the index for area *α* during period *p* in year *y*. To calculate a confidence interval, the standard error of the mean is first calculated as

(5) $$s_{a,p,y}/\sqrt{n}$$

where *n* is the number of recording sites. This quantity expectedly follows the t-distribution with (*n* − 1) degrees of freedom. Thus, a confidence interval of level (1 − *α*) for an estimated index for year y is calculated as

(6) $$Q_{a,p,y}\;\pm \;t_{n-1}(\alpha /2)\;\cdot \;\frac{s_{a,p,y}}{\sqrt{n}}$$

Here, *t_n_*_−1_(*α*/2) is the upper *α*/2 quantile of the t-distribution with (*n* − 1) degrees of freedom. The indices *Q_a_*_,*p*,*y*_ and the corresponding confidence intervals may be expressed as percentages by multiplying by 100%. We consider the change significant when the confidence interval does not cross 100 since each year is compared to 2018 (100 [95% CI: 100–100]).

## 3. Results

**Figure 2:**
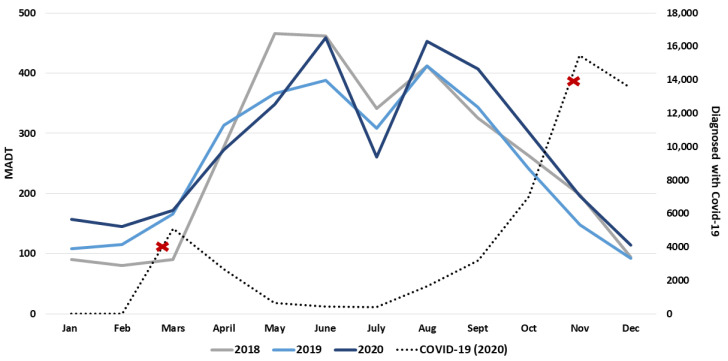
Monthly national bike traffic from January 2018 to December 2020 highlighting the monthly number of individuals diagnosed with COVID-19 (2020). Red crosses illustrate implementation of national strategies to combat COVID-19. MADT stands for monthly average daily traffic.

From 2018 to 2020, the national index indicates a significant 11% increase in the number of counted cycle trips. The national index was 97 (94–100) in 2019 and 111 (106–115) in 2020 (see Table 2 for details). In 2020, more passing cyclists were counted during winter and autumn (Figure 2). In Norway, there seems to be a consistent seasonal pattern in which the number of counted cycle trips is threefold larger in May and June compared with January. A further drop in counted cycle trips occurs in July (summer holiday) followed by a second peak in August.

### Regional and Local Trends in Bike Traffic

**Figure 3:**
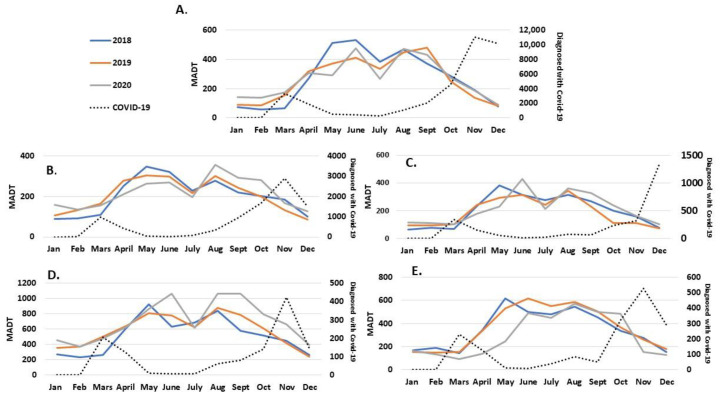
Regional monthly average daily traffic from 2018 to 2020 highlighting the monthly number of individuals diagnosed with COVID-19 (2020). (**A**) Eastern Norway, (**B**) Western Norway, (**C**) Mid Norway, (**D**) Sothern Norway, (**E**) Northern Norway. MADT stands for monthly average daily traffic.

We found regional differences in trends of counted cycle trips. Southern and Western Norway had a continuous increase in counted cycle trips, with Southern Norway having a 23% (123, 107–140) increase over the last three years. The only region with a decrease in counted cycle trips was Northern Norway, where the number of counted cycle trips decreased by 8% from 2018 to 2020 (92, 72–112). Both Northern and Southern Norway had a 17 to 20% uncertainty mainly due to the low number of included counters (see Table 2 for details). For Western and Mid Norway, there was a statistically significant increase of 11% over the last three years, with small regional differences in patterns (Figure 3). We observed large differences in local trends over the last three years (Table 2). In Førde, Western Norway, the level of counted cycle trips increased by 4% from 2018 to 2020; however, the confidence interval indicates the uncertainty of the result. The largest local increase was observed in Drammen (Eastern Norway) and Kristiansand (Southern Norway) with 153 and 23% increases, respectively. However, the increase was only statistically significant for Kristiansand.

## 4. Discussion

The national bike traffic index suggests that the number of cycle trips in Norway increased significantly by 11% from 2018 to 2020. However, we observed regional and local differences. The differences between regions and local areas highlight the advantages of indices of smaller geographical areas. Furthermore, most interventions are local, and a local index is a valuable tool to evaluate these interventions. At a national level, we observed seasonal differences with the highest level of counted cycle trips occurring from May to August, with a consistent period of fewer trips in the autumn and winter months. Ninety-three per cent of the included counters have a commuter or a commuter-mixed traffic pattern. Therefore, the index mainly describes the trends of commuter cycling, and thus the index may be defined as an index of commuter cycling. The Norwegian government is continuing the strategy of increasing the level of commuter cycling in highly populated areas [12]. The present national index and local indices may directly evaluate the national, regional, and local strategies and measures.

The aim of the present study was to develop a bike traffic index and describe the national, regional, and local trends in counted cycle trips in Norway. From a short, random sample for all counters, the calculation of traffic patterns indicates that a majority of counters describe trends in commuter cycling. The results must be integrated with knowledge of local, regional, and national strategies and actions to promote cycling to more precisely describe factors possibly affecting the trend. However, the national trend in counted cycle trips was a small national decrease in counted cycling trips in 2019 followed by a rather large increase in 2020. We are not aware of any national campaigns in the last years to increase commuter cycling, but there is a small yet steady increase in cycling-friendly infrastructure in accordance with the national transport plans [1,13]. In 2018, 199 km of new cycle-friendly infrastructure (including cycle paths and combined pedestrian and cycle paths) was finalized, while the corresponding numbers for 2019 and 2020 were 173 and 322 km, respectively [28]. Due to a national reorganization of municipalities and counties in 2020, data below the national level cannot be derived from Statistics Norway. Several studies [19,29,30,31,32] have observed positive associations and effects between cycling-friendly infrastructure and commuter cycling [11,16,19]. In 13 European cities with low to medium cycling levels, a linear relationship (R^2^ = 0.8) has been observed between metres of cycle-friendly infrastructure per citizen and bike mode share [11]. Others have found that cycle-friendly infrastructure explains one-third of the variation in commuter cycling rates [17,33]. However, even with perfect conditions for commuter cycling, some individuals will still choose a mode of transportation other than a bicycle [11]. It is plausible that the significant increase in counted bicyclists is a result of more cycling-friendly infrastructure, but no causal conclusion can be drawn from the present study [34]. Since the importance of the built environment (i.e., cycle-friendly infrastructure) is likely mediated by personal factors, infrastructure alone is not sufficient to increase cycling rates [20]. Furthermore, building new cycle-friendly infrastructure is expensive. However, from a 25-year perspective, the health benefits are more than five times larger than the cost of building the cycle-friendly infrastructure [10,11]. In terms of health benefits at a population level in a country with cycle-friendly infrastructure, increased rates of cycling are 21 and 238 times higher than the risk of injuries and mortality caused by cycle accidents, respectively [9]. From a socioecological perspective, changes in behaviour (in this context, cycling) are more likely to occur when interventions implement actions on multiple levels, from the individual level to community and policy levels [34]. Due to the complexity of behaviour change, increased counted cycle trips in Norway during the last three years may be led by other factors than changes in the built environment.

Another factor that may have affected travel habits in Norway in 2020 is the COVID-19 pandemic. In Norway, there was a national lockdown during spring 2020 and a second lockdown in late autumn 2020. Although the second lockdown was a national strategy, the local implementation varied. The national lockdown included closure of preschools, and all levels of schools provided remote learning. All shops, restaurants, and services were closed, and remote work was standard for all citizens whenever possible. Social contact was guided towards an absolute minimum. After the lockdown, Norwegian citizens were encouraged to minimize the use of public transport (i.e., bus, train, and tram), only travel when needed, keep social contact at a minimum, and work remotely when possible. The promotion of not using public transport may have led to an increase in the use of micro mobilities [35] and private cars [35,36]. The national index indicates that a higher volume of counted cycle trips may be a result of reduced use of public transport as observed in both European and American cities [37]. However, the national index only describes total cycling. The calculated traffic pattern indicates that included counters mainly count commuter traffic; however, the increase may also have been an increase in recreational cycling. In European cities, a total increase of 8% from 2019 to 2020 has been observed [37], while in a worldwide cross-sectional study, the proportion of cyclists has increased from 8 to 26% [36]. Some studies report that the largest increase is seen on weekends, indicating an increase in recreational cycling [37,38].

### 4.1. The Present Bike Traffic Index Compared to the National Travel Survey

The bike traffic index supplements the Norwegian travel survey. Together they provide reliable data to evaluate strategies at the local, regional, or national level. From 1985 to 2014, the travel survey was conducted every fourth year. Since 2016, the travel survey has been published annually and conducted by NRPA. While the travel survey is conducted annually, the index provides monthly and annual data with a much larger sample size. The last two travel surveys have had 47,806 and 110,672 respondents, with a 5% share of cyclists [39,40]. The present bike traffic index covers an area of more than 1.2 million people and thus is likely to be more sensitive regarding changes in cycling habits.

### 4.2. Sensitivity Analyses

The present index is weighted for population density in accordance with the Danish bike traffic index [23]. The index could possibly be weighted for other factors, such as type of road, weather, type of day, traffic pattern, and cycle infrastructure [40]. For the present model, multiple models built on parameters conserving mean counts, population density, distance between counters, and a counter’s number of operative days were tested. The variance between the models was 4.1% (see Appendix A). Therefore, the present index was only weighted only for population density around the counter.

### 4.3. Strengths and Limitations

The present bike traffic index is a measure of counted passings over a stationary counter and does not necessarily have the same trend as travel surveys where one examines either the proportion of cycle trips out of the total number of trips or the proportion of cyclists. The present bike traffic index describes the trends in counted cycle trips where an increased number of trips may reflect that more people are cycling or that a person cycles more frequently. Given the ecological design of the present study, one should be aware of the possibilities of ecological fallacy since the study is not based on individual data. The present index describes counted trips with indications of cycling mode based on calculation of traffic pattern forming a short, random period and thus describing total cycling with *indications* of commuter cycling before the COVID-19 pandemic. If Norwegian travel patterns follow European and American mobility trends during the COVID-19 pandemic [37], it is possible that the observed increase in counted cycle trips is reflecting more recreational cycling rather than commuter cycling.

It has been argued that bike traffic indices must have at least one of each day of the week in each month to have sufficient data quality [40]. Furthermore, the error may be minimized by using factors that take weather into account [40]. In the present bike traffic index, we handled missing data at a daily level by interpolating by linear regression, where the missing value was set to the mean of the nearest valid values next to the missing value. Furthermore, only pairs of months with valid data were included in the index.

Unfortunately, the present national bike traffic index is mainly based on counters in urban areas. However, in Norway there are large areas with rural populations. The index has the limitation of not describing rural bike traffic trends due the lack of counters in rural areas. For urban areas, the present bike traffic index has several advantages for detecting changes. Moltved et al. [23] highlight three specific advantages for bike indices with similar methods as the present index. First, the bike counters include the actual number of passing cyclists independent of residence, age, or recall bias. Second, the counter’s location is precisely described, and third, continuous counting results in a model which is sensitive to actual changes. Furthermore, the present bike traffic index is a robust yet dynamic model. The present bike traffic index uses the sum of counted trips from local indices in both national and regional indices. We have therefore developed a model which enables the inclusion of both new counters and local indices when more counters are operative.

## 5. Conclusions

The present study describes the methods of a sensitive bike traffic index at local, regional, and national levels from 2018 to 2020 and was intended to follow trends in counted trips for years. The bike traffic index of counted cycle trips has described the 2018 level and trends in Norway over subsequent years. Nationally, we observed a significant 11% increase in counted cycle trips. However, local and regional indices indicate local differences. The indices may indicate the possible related effects of local to national cycling strategies and constitute a sensitive tool for monitoring changes in cycling habits. Calculations indicate that most counters are mainly passed by commuter cyclists, but the index itself only describes trends in total counted trips. No conclusion regarding possible explanations of the significant increase in counted trips can be drawn from this study. However, the trend observed is in accordance with the literature regarding the increased metres of cycle-friendly infrastructure and how the COVID-19 pandemic affected travel habits globally in 2020.

## Figures and Tables

**Table 1:** Number of counters and population density at the local and regional level.

Region	Local Area	Number of Counters	Mean Population Density
**Southern Norway**		3	24,780
	Kristiansand	3	24,780
**Northern Norway**		3	23,474
	Bodø	2	16,876
	Tromsø	1	30,073
**Mid Norway**		6	20,964
	Steinkjer	2	10,245
	Trondheim	2	32,547
	Verdal	2	8519
**Eastern Norway**		48	29,670
	Hamar	1	20,252
	Elverum	1	8012
	Oslo	6	93,176
	Sande	1	3618
	Porsgrunn	6	10,043
	Skien	14	18,195
	Tønsberg	4	16,204
	Drammen	2	25,865
	Fredrikstad	3	25,325
	Moss	5	10,512
	Sarpsborg	5	13,970
**Western Norway**		29	15,148
	Bergen	12	25,113
	Flora	3	4203
	Førde	8	5245
	Egersund	2	4221
	Kristiansund	1	10,982
	Bø	1	4266
	Haugesund	1	18,368
	Stavanger	1	840
**Norway**		89	22,631

**Table 2:** National, regional, and local weighted * indices with a 95% confidence interval from 2018 to 2020.

	Number of Counters	2018	2019	2020
**National**	89	100	97.0 (94.1–99.8)	111.0 (106.2–115.1)
**Regional**				
Southern Norway	3	100	103.5 (101.2–105.7)	123.2 (106.5–140.0)
Northern Norway	3	100	104.8 (61.3–148.4)	91.7 (71.6–111.8)
Western Norway	29	100	102.0 (96.5–107.6)	111.3 (101.4–120.9)
Eastern Norway	48	100	93.6 (89.6–97.3)	111.3 (104.5–117.0)
Mid Norway	6	100	94.2 (85.7–102.6)	103.4 (95.7–111.1)
**Local**				
Kristiansand	3	100	103.5 (101.2–105.7)	123.2 (106.6–140.0)
Elverum	1	100	87.8	78.0
Hamar	1	100	91.2	108.8
Kristiansund	1	100	108.6	106.9
Bodø	2	100	106.7 (−78.9–292.2)	89.3 (24.4–154.2)
Oslo	6	100	94.3 (87.5–100.6)	118.8 (91.7–144.3)
Egersund	2	100	101.5 (79.5–123.6)	108.4 (81.7–135.1)
Tromsø	1	100	96.1	100.7
Steinkjer	2	100	91.3 (51.7–130.9)	113.6 (49.6–177.6)
Trondheim	2	100	94.2 (1.2–187.1)	100.9 (96.1–105.6)
Verdal	2	100	96.6 (95.6–97.6)	113.0 (2.5–223.5)
Porsgrunn	6	100	87.1 (80.4–93.8)	104.9 (95.0–114.9)
Sande	1	100	93.4	119.6
Skien	14	100	95.3 (90.4–100.2)	106.2 (100.6–111.8)
Tønsberg	4	100	97.1 (92.1–102.0)	109.2 (101.6–116.8)
Bergen	12	100	103.8 (92.4–115.5)	117.9 (99.8–136.1)
Kinn	3	100	95.0 (91.8–98.1)	86.5 (72.9–100.2)
Førde	8	100	104.0 (92.1–115.9)	104.6 (83.8–125.4)
Drammen	2	100	120.9 (−935.9–1177.7)	253.8 (−150.5–658.0)
Fredrikstad	3	100	68.1 (9.6–126.8)	81.4 (−12.5–175.5)
Moss	5	100	91.8 (83.0–100.5)	106.1 (88.9–123.4)
Sarpsborg	5	100	92.4 (89.7–95.2)	108.8 (101.1–116.6)
Stavanger	1	100	84.0	120.8
Haugesund	1	100	93.2	96.0
Bø	1	100	96.1	98.7

* Weighted for population density.

## Data Availability

Publicly available datasets were analyzed in this study. This data can be found here: www.trafikkdata.no (accessed on 7 June 2021).

## Appendix A. Sensitivity Models of the National Bike Traffic Index

**Table A1:** Sensitivity mdels * of the national bike traffic index.

Year	Model 1	Model 2	Model 3	Model 4	Model 5	Model 6	Model 7	Model 8
2018	100.0	100.0	100.0	100.0	100.0	100.0	100.0	100.0
2019	95.4	94.6	95.4	94.7	93.9	97.2	93.1	94.5

* All models are based on 79 counters operative from 1 July 2017.

**Model 1:** The model is the mean of all counts (*C*) divided by the total number of counters: 79 (*T*).

(A1) $$=\frac{C_{1}+C_{2\ldots \;}+C_{n}}{T_{n}}$$

**Model 2:** Mean of annual average daily trips (*AADT*) per local index (*l*) divided by the number of indices (*l_n_*).

(A2) $$=\frac{\bar{AADTl_{1}\;}+\;\bar{AADTl_{2\ldots }}+\;\bar{AADTl_{n}\;\;}\;}{l_{n}}$$

**Model 3:** Weighted number of counters within the local index. When there are one or two counters, the local *AADT* is multiplied by 1; when there are three or four counters, the local *AADT* is multiplied by 2; and with more than five counters, the index is multiplied by 3.

**Model 4:** Weighted percentage of volume *AADT* per local index.

(A3) $$=\frac{\bar{AADTl_{1}}}{AADTl_{1}+AADTl_{2\ldots \;}+AADTl_{n}}+\frac{\bar{AADTl_{2\ldots }}}{AADTl_{1}+AADTl_{2\ldots \;}+AADTl_{n}}+\frac{\bar{AADTl_{n}}}{AADTl_{1}+AADTl_{2\ldots \;}+AADTl_{n}}$$

**Model 5:** Weighted population density per municipality, where *Pm* = total population, *pm* = population in local index.

(A4) $$=\frac{pm\bar{\;AADTl_{1}}}{Pm}+\frac{pm\bar{\;AADTl_{2\ldots }}}{Pm}+\frac{pm\bar{\;AADTl_{n}}}{Pm}$$

**Model 6:** Weighted population in 5-km grid per counter, where *Pg* = total population in all grids with counter, *Pg* = population in grid with counter, and *C* = counter.

(A5) $$=\frac{pg\bar{\;AADTC_{1}}}{Pg}+\frac{pg\bar{\;AADTC_{2\ldots }}}{Pg}+\frac{pg\bar{\;AADTC_{n}}}{Pg}$$

**Model 7:** Weighted population in 5-km grid per local index, where *Pg* = total population in all grids with counter, *Pg* = population in grid with counter, and *l* = local index.

(A6) $$=\frac{pg\bar{\;AADTl_{1}}}{Pg}+\frac{pg\bar{\;AADTl_{2\ldots }}}{Pg}+\frac{pg\bar{\;AADTl_{n}}}{Pg}$$

**Model 8:** Weighted distance (> or <4.9 km; Average length of daily trips in Norway) between counters in local index, where *d* = average distance between counters > 1 = 1.

(A7) $$=\frac{\bar{d\;AADTl_{1}}}{l_{1}}+\frac{\bar{d\;AADTl_{2\ldots }}}{l_{2\ldots }}+\frac{\bar{d\;AADTl_{n}}}{l_{n}}$$
